# Differential roles for CLA-1L and UNC-10 in endosomal maturation and peptide release at *C. elegans* synapses impacting lifespan

**DOI:** 10.3389/fmolb.2025.1675073

**Published:** 2026-01-08

**Authors:** Mia Krout, Elena Miciulis, Phong Q. Lai, Janet E. Richmond

**Affiliations:** Biological Sciences, College of Liberal Arts and Sciences, University of Illinois Chicago, Chicago, IL, United States

**Keywords:** RIM, cla-1, C.elegans, endosome maturation, neuropeptide release

## Abstract

*Caenorhabditis* *elegans* encode two synaptic proteins linked to the Rim/Piccolo/Fife-family, through conserved motifs: 1) Clarinet (CLA-1), has 3 isoforms (short(S), medium(M) and long(L)) that are anchored at the active zone through a common C-terminal domain and 2) UNC-10/Rim that is also highly enriched at the presynaptic density. Both the *cla-1* and *unc-10* mutants have demonstrable effects on synaptic transmission and in combination produce a synergistic impact that virtually eliminates synaptic transmission and that has yet to be fully understood. Recently, CLA-1L and UNC-10 were shown to differentially regulate key active zone components, culminating in reduced Ca^2+^ channels and UNC-13 levels, but these changes cannot account for the severity of the release defects in the double mutants. CLA-1L extends far beyond the synaptic active zone and has been implicated in recycling of the key autophagy protein ATG-9. In this study, we show that *cla-1L* and *unc-10* mutants negatively impact proteins involved in endocytic processing (ITSN-1 and AP-2) and endolysosomal maturation (RAB-5 and RAB-7). These abnormalities correlate with an accumulation of synaptic pleiomorphic vesicles by EM, in both *cla-1L* and *unc-10* mutants. In addition, *unc-10* mutants accumulate dense core vesicles, due to a dramatic reduction in neuropeptide release. These observations are accompanied by significant decreases in lifespan in both *cla-1L* and *unc-10* mutants, which are exacerbated in the double mutants. Together these data suggest that the cumulative effects on synaptic transmission that result from distinct roles of CLA-1L and UNC-10 have an impact on survival.

## Introduction

In eukaryotic cells, endocytosis and autophagy are interconnected processes that work together to maintain cellular homeostasis. A recent genetic screen revealed that *Caenorhabditis elegans* Clarinet (CLA-1), a synaptic protein related to active zone proteins; RIM, Piccolo, and Fife-1, plays a role in the neuronal autophagy pathway (Xuan et al., 2023). Specifically, loss of the long isoform - CLA-1L, disrupts the presynaptic localization of ATG-9, phenocopying endocytic mutants, and leads to defects in autophagosome formation. These data implicate CLA-1L in coupling of the endosomal and autophagic pathways.

The endocytic arm of this system involves the internalization and processing of extracellular and membrane-associated cargo through a series of endo-lysosomal organelles, each characterized by distinct molecular markers, lumenal content, and functions. Endocytosis typically begins with the budding of vesicles from the plasma membrane. While clathrin-mediated endocytosis is a well-characterized route, clathrin-independent pathways such as activity-dependent bulk endocytosis (ADBE) and ultrafast endocytosis (UFE) also contribute to membrane retrieval (Bouhamdani et al., 2021; Watanabe et al., 2014; Wu et al., 2014). Intersectin (ITSN-1) is one of several proteins that coordinate these early stages of the endosomal pathway. ITSN-1 is a highly conserved SH3 containing protein that is enriched at the synaptic endocytic zone and associates with early endosomes via AP-2 interaction, acting as a scaffold for dynamin, EPS-15/EHS-1, and Synaptojanin—proteins known to play key roles in synaptic vesicle recycling and endosomal sorting (Haucke et al., 2011; Rose et al., 2007; Wang et al., 2008; Watanabe et al., 2014). AP-2 is involved in cargo recognition, clathrin-cage assembly and recruitment of the scission machinery, during both clathrin-mediated and bulk endocytosis, thereby aiding in vesicle recycling (Kononenko et al., 2014; Traub, 2003). Newly formed endocytic vesicles can fuse with early endosomes (EEs), where internalized cargo can be sorted via complexes such as ESCRT or SNX-1/RME-8 before they mature (Norris et al., 2017; Rojas et al., 2007). Endosome maturation is necessary for the delivery of endocytosed materials to lysosomes for degradation. Small GTPases (Rabs) regulate the maturation of EEs to late endosomes (LEs) (Bucci et al., 1992; Wandinger-Ness and Zerial, 2014). EEs are labelled by RAB-5, which regulates the fusion of newly endocytosed vesicles with existing EEs, as well as homotypic fusion between EEs (Casanova and Winckler, 2017; Gorvel et al., 1991; Stenmark et al., 1994). LEs are labelled by RAB-7, which regulates their homotypic fusion and fusion of LEs with lysosomes (Casanova and Winckler, 2017; Wandinger-Ness and Zerial, 2014). For an endosome to mature from an EE to LE, RAB-5 must be replaced by RAB-7 in a well-characterized process referred to as Rab5/Rab7 conversion (Casanova and Winckler, 2017; Deinhardt et al., 2006; Poteryaev et al., 2010). In eukaryotic cells, both endocytic and autophagic compartments fuse with lysosomes - a point of convergence in cellular homeostasis.

Autophagy is a tightly regulated catabolic process that degrades and recycles cytoplasmic components, such as damaged organelles and misfolded proteins, to maintain cellular homeostasis (Bento et al., 2016; Boland et al., 2008; Maday and Holzbaur, 2014; Shibutani and Yoshimori, 2013). A key regulator of this process is ATG9 (autophagy related gene 9), the only transmembrane autophagy protein, which plays a critical role in delivering membrane required for autophagosome biogenesis (Judith et al., 2019; Orsi et al., 2012; Park et al., 2023; Stavoe et al., 2016). In neurons, ATG9 is trafficked from the trans-Golgi network to synapses where it cycles through the exo-endocytic cycle in an activity-dependent manner. In *C. elegans,* endocytic mutations that disrupt this cycle cause abnormal ATG-9 aggregates in clathrin-rich subdomains, demonstrating coupling between endocytosis and autophagy (Yang et al., 2022). Disruption of ATG-9 trafficking impairs autophagosome formation and is linked to human neurodegenerative diseases, emphasizing the importance of coordinated endo- and autophagic-lysosomal pathways in maintaining neuronal health (Judith et al., 2019; Orsi et al., 2012; Yang et al., 2022).

Here we used the *cla-1* long isoform mutant, *cla-1(ok560)*, and the *unc-10* null mutant *unc-10(md1117)*, hereafter referred to as *cla-1L* and *unc-10*, respectively, to investigate how CLA-1L and UNC-10, individually and in combination, regulate key components of the endo-lysosomal pathway and explore the functional consequences of their loss.

## Results

### Loss of CLA-1L and UNC-10 disrupt ITSN-1 and AP-2, two components of the endocytic machinery

**FIGURE 1:**
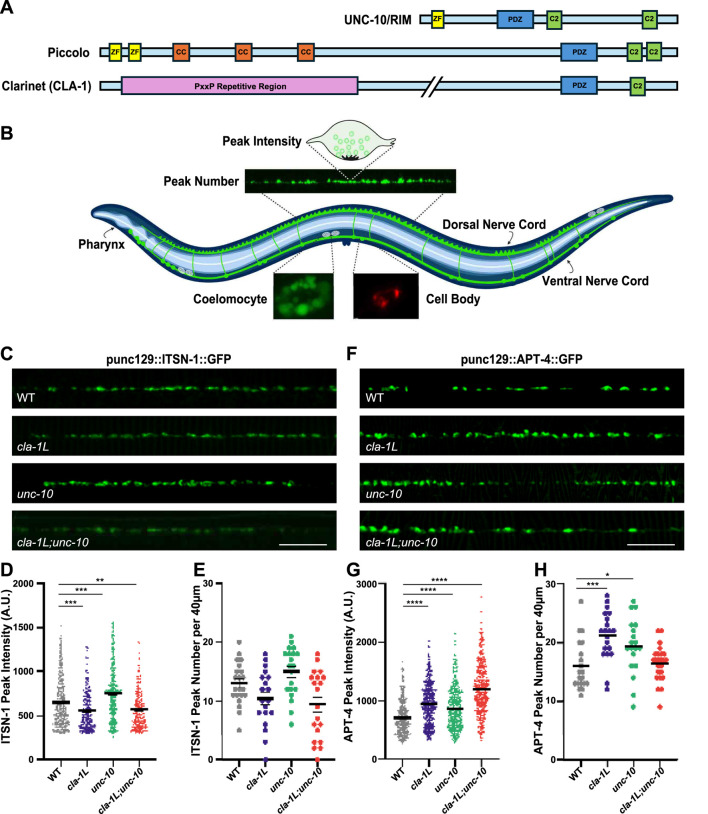
Loss of CLA-1L and UNC-10 disrupt the synaptic localization of endocytic proteins, ITSN-1 and AP-2. **(A)** Schematic depicting the relative size and functional domains of RIM family proteins, RIM/UNC- 10, Piccolo (PCLO) and full-length Clarinet isoform (CLA-1L). **(B)** Schematic representation of an adult *C. elegans* worm showing the position of dorsal and ventral nerve cords that innervate cuticle attached dorsal and ventral body wall muscles. Upper inset shows a region of the dorsal nerve cord from which all synaptic fluorescent puncta in this study were analyzed. Peak number refers to the number of synaptic puncta per 40 μm and peak intensity refers to the fluorescence value of individual puncta. Also shown is the position of coelomocytes and ventrally located cell bodies that project to the dorsal nerve cord. Left lower inset shows an example of a coelomocyte labeled with NLP-21 used as a proxy for peptide secretion; right lower inset shows a neuronal cell body expressing lysosomal protein, CTNS-1. Created in BioRender. Lai, P. (2025) https://BioRender.com/y2rqru0. **(C)** Representative images of dorsal nerve cords of WT and cla-1L, unc-10 and cla-1L; unc-10 mutant animals expressing ITSN-1:GFP. Scale bar 10 μm. **(D,E)** Graphs displaying quantitative analysis of **(D)** fluorescent peak intensity and **(E)** peak number of ITSN-1:GFP in Control and mutant animals. **(F)** Representative images of dorsal nerve cords of Control and cla-1L, unc-10 and cla-1L; unc-10 mutant animals expressing APT-4:GFP as a fluorescent marker of the alpha subunit of AP-2. Scale bar 10 μm. **(G,H)** Graphs displaying quantitative analysis of **(G)** fluorescent peak intensity and **(H)** peak number of APT-4:GFP in WT and mutant animals. Statistical analysis: One-way ANOVA with Kruskal–Wallis with Dunn’s test for multiple comparisons; *p < 0.05, **p < 0.01, p < 0.001, p < 0.0001 results showing mean and S.E.M.

CLA-1L is a 9,000-amino-acid protein, anchored via its C-terminal at the presynaptic density, and extends an unstructured N-terminal domain into the periactive zone—a region associated with clathrin-mediated endocytosis (Krout et al., 2023; Xuan et al., 2017; Xuan et al., 2023). In the CLA-1L N-terminal sequence, we have identified a repetitive region that contains approximately 20 PxxP motifs, which are potential SH3-binding domains that could interact with endocytic proteins (Figure 1A). A recent study found that both *cla-1L* mutants and *itsn-1* mutants disrupt the presynaptic distribution of ATG-9, and this disruption was enhanced in *cla-1L;itsn-1* double mutants (Xuan et al., 2023). We therefore asked whether fluorescently labeled ITSN-1 (ITSN-1:GFP) was affected by the loss of CLA-1L, specifically in a subset of dorsal cholinergic neuromuscular junctions (NMJs) using the *unc-129* promoter, which allows individual synaptic puncta to be clearly discernible (Figure 1B). We observed a significant decrease in the peak fluorescence intensity of ITSN-1 in *cla-1L* mutants, whereas peak number along the dorsal nerve cord (DNC) was unchanged (Figures 1C–E). Given that the short and medium *cla-1* isoforms, which share a common C-terminal with CLA-1L, are still expressed in *cla-1L* mutants, this suggests that an interaction between CLA-1L and ITSN-1 likely occurs via the unique proline-rich N-terminal of CLA-1L (Krout et al., 2023; Xuan et al., 2017). As UNC-10 lacks a proline-rich domain, we would not expect loss of UNC-10 to disrupt the synaptic localization of ITSN-1. This was confirmed, and in fact *unc-10* mutants exhibited increased ITSN-1 peak fluorescence intensity (Figures 1C–E). Analysis of the *cla-1L;unc-10* double mutants showed a decrease in peak fluorescence, similar to that of *cla-1L* mutants alone, suggesting that CLA-1L is the necessary component for proper ITSN-1 localization.

Given that ITSN-1 is recruited by the adaptor protein complex 2 (AP-2), we next asked whether the AP-2 complex (visualized through its fluorescently tagged alpha subunit, APT-4:GFP) is affected by the loss of CLA-1L and the associated reduction in synaptic ITSN-1. Interestingly, in *cla-1L* mutants we observed a significant increase in both the peak fluorescence intensity and number of peaks expressing APT-4 (Figures 1F–H). We also saw a similar increase in APT-4 peak fluorescence intensity and number in *unc-10* mutants. In previous studies, synapse density in *cla-1L* and *unc-10* mutants (assessed by number of fluorescent peaks using multiple endogenously tagged synaptic proteins) was not found to be increased, suggesting that the additional APT-4 peaks detected in the single mutants may represent abnormal APT-4 localization (Krout et al., 2023; Xuan et al., 2017). There was a similar increase in APT-4 peak fluorescence intensity in the *cla-1L;unc-10* double mutants, though the observed increase in peak number did not reach statistical significance. Since ITSN-1 is differentially impacted in these mutants, the APT-4 data suggest that there may be different underlying causes for the changes in ITSN-1 fluorescent peak intensity and distribution in *cla-1L* and *unc-10* mutants.

### The synaptic distribution of endosomal markers, RAB-5 and RAB-7, is perturbed in *cla-1L* and *unc-10* mutants

**FIGURE 2:**
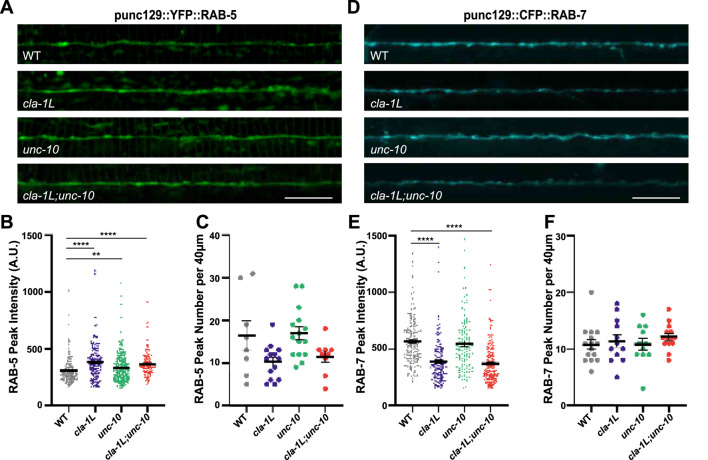
The abundance of endosomal proteins, RAB-5 and RAB-7, is altered upon loss of CLA-1L and UNC-10. **(A)** Representative images of dorsal nerve cords of WT and cla-1L, unc-10 and cla-1L; unc-10 mutant animals expressing YFP::RAB-5. Scale bar 10 μm. **(B,C)** Graphs displaying quantitative analysis of **(B)** fluorescent peak intensity and **(C)** peak number of YFP::RAB-5 in WT and mutant animals. **(D)** Representative images of dorsal nerve cords of WT and cla-1L, unc-10 and cla- 1L; unc-10 mutant animals expressing CFP::RAB-7. Scale bar 10 μm. **(E,F)** Graphs displaying quantitative analysis of **(E)** fluorescent peak intensity and **(F)** peak number of CFP::RAB-7 in WT and mutant animals. Statistical analysis: One-way ANOVA with Kruskal–Wallis with Dunn’s test for multiple comparisons; **p < 0.01, *p < 0.0001 results showing mean and S.E.M.

Once vesicles have been internalized they are uncoated and fuse with early endosomes, which are labelled by RAB-5. Given the observed changes in vesicle endocytic proteins, we next examined YFP::RAB-5 in the same synapses of *cla-1L* and *unc-10* and the double mutants. RAB-5 fluorescence peak intensity but not peak number was significantly increased in *cla-1L* mutants, suggesting that RAB-5 accumulates at these synapses (Figures 2A–C). In *unc-10* mutants there was also a significant increase in RAB-5 peak intensity, though to a lesser extent than *cla-1L* mutants. In the *cla-1L;unc-10* double mutants the intensity of the RAB-5 peaks was most similar to that of *cla-1L* mutants.

As RAB-5 accumulates at the synapses of *cla-1L* and, to a lesser degree, *unc-10* mutants, we hypothesized that endosomal maturation may be disrupted. To test this, we examined CFP::RAB-7 levels. While peak number was not impacted in either single or double mutants, the intensity of RAB-7 fluorescent peaks was significantly decreased in *cla-1L* and *cla-1L;unc-10* mutants, with no change in the *unc-10* single mutants (Figures 2D–F). The lack of an effect on RAB-7 peaks in *unc-10* mutants was not entirely surprising given its small impact on RAB-5 levels. Taken together, these observations suggest that CLA-1L impacts the RAB-5 to RAB-7 conversion step of endosome maturation, and while loss of UNC-10 has some effect on the endosomal pathway, its role appears to be minor.

### Pleiomorphic vesicles accumulate at NMJ synapses with loss of CLA-1L and UNC-10

**FIGURE 3:**
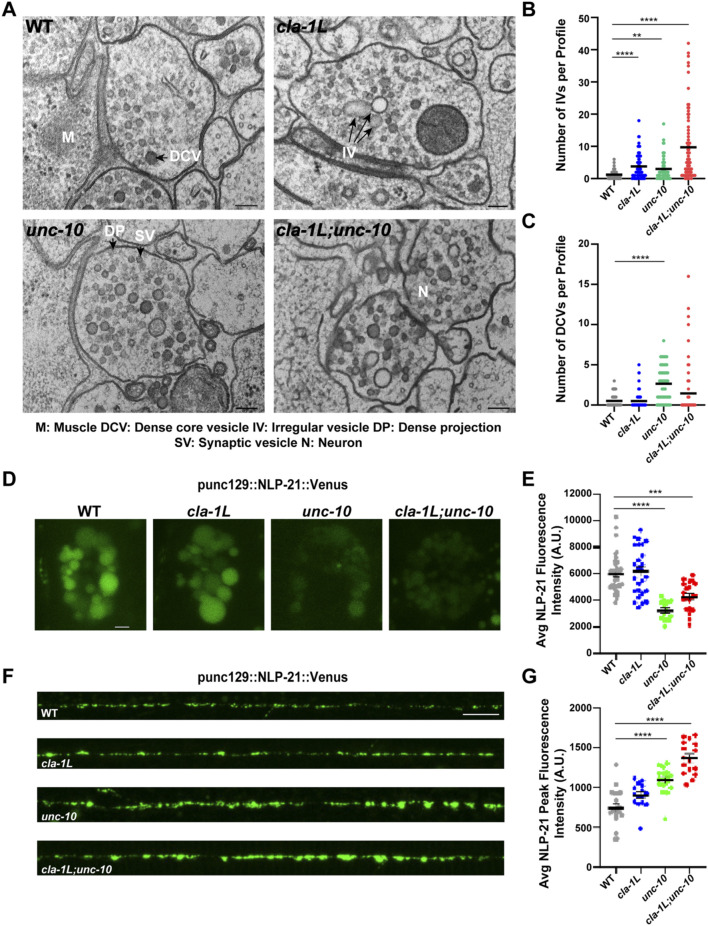
CLA-1L and UNC-10 independently contribute to the vesicle cycle at synapses. **(A)** Representative electron micrographs of NMJ synapses taken in WT (top left) and cla-1L (top right), unc-10 (bottom left) and cla-1L; unc-10 (bottom right) mutant animals. Text and black arrows point to an example of a dense projection (DP) synaptic vesicle (SV), a dense core vesicle (DCV), and irregular vesicles (IV). Scale bar 100 nm. **(B)** Graph displaying quantitative analysis of irregular clear or light grey vesicles at NMJ synapses in WT and mutant worms. Vesicles are quantified per synaptic profile. A synaptic profile is defined as a section that contains a DP, an area containing a large number of proteins involved in synaptic release, and the two flanking sections on either side of the DP. **(C)** Graph displaying quantitative analysis of dense core vesicles in NMJ synapses of WT and cla-1L, unc-10 and cla-1L; unc-10 mutant animals. Vesicles are quantified per synaptic profile. A synaptic profile is defined as a section that contains a DP and the two flanking sections on either side of the DP. Total data analyzed for EM: WT = 83 profiles from 12 synapses; cla-1L = 74 profiles from 10 synapses; unc-10 = 64 profiles from 9 synapses; cla-1L; unc-10 = 75 profiles from 11 synapses. **(D)** Representative images of NLP-21:Venus levels in coelomocytes of WT and cla-1L, unc-10 and cla-1L; unc-10 mutants. Scale bar 2 μm E) Graph displaying quantitative analysis of the summed coelomocyte NLP-21:Venus fluorescence intensity in WT and mutant animals. **(F)** Representative images of dorsal nerve cord NLP-21:Venus expressed in cholinergic synapses. Scale bar 10 μm G) Graph displaying quantitative analysis of the summed coelomocyte NLP-21:Venus fluorescence intensity in WT and mutant animals. Statistical analysis: One-way ANOVA with Kruskal–Wallis with Dunn’s test for multiple comparisons; **p < 0.01, ***p < 0.001, *p < 0.0001 results showing mean and S.E.M.

Based on the changes in endocytic and endosomal markers observed above, we asked whether *cla-1L* and *unc-10* mutants exhibit signs of synaptic abnormalities in endosomal structures. To examine this, we performed high-pressure freeze fixation and freeze substitution-electron microscopy (HPF/FS-EM) (H. Liu et al., 2021; Weimer, 2006). NMJ micrographs from *cla-1L* and *unc-10* mutants revealed a significant increase in the number of large pleiomorphic vesicles: both irregular and spherical clear vesicles, often aggregating via filamentous connections with other vesicles (Figures 3A,B; Supplementary Figure S1A). Strikingly, *unc-10* mutants also accumulated dense core vesicles (DCVs) of varying size, in contrast to *cla-1L* (Figures 3A–C; Supplementary Figure S1A). Furthermore, the *cla-1L;unc-10* double mutants accumulated both vesicle types, suggesting that CLA-1L and UNC-10 differentially regulate the vesicle composition at the NMJ.

UNC-10 has not previously been reported to impact DCVs, given the novelty of this finding, we employed a well-established assay for peptide secretion to determine whether the accumulation of DCVs in *unc-10* mutants reflects defective DCV fusion (Sieburth et al., 2006). In this assay, levels of a fluorescently tagged peptide (NLP-21:Venus) released from motor neurons are measured in coelomocytes—endocytic cells responsible for the uptake of secreted proteins from the pseudocoelom of the worm. Thus, coelomocyte NLP-21 levels act as a proxy for neuronal NLP-21 release. This analysis revealed a dramatic reduction in coelomocyte NLP-21 fluorescence in *unc-10* and *cla-1L;unc-10* mutants, consistent with impaired peptide secretion from NMJs (Figures 3D,E). As predicted by the normal number of DCVs observed in *cla-1L* mutant synapses, NLP-21 fluorescence levels were unaffected, indicating the peptide release defect is specific to *unc-10* mutants. To reinforce this conclusion, we conducted two control experiments. First, we measured NLP-21 levels in the DNC and found a corresponding increase in fluorescence peak intensity in *unc-10* and *cla-1L;unc-10* double mutants consistent with impaired release, whereas *cla-1L* mutants were unaffected (Figures 3F,G). Second, to ensure that the reduced peptide levels in *unc-10* and *cla-1L;unc-10* coelomocytes were not the result of defective endocytic activity we injected Texas Red-BSA into the pseudocoelom and measured levels of coelomocyte uptake after 20 min. Texas Red accumulation was similar in all strains, demonstrating that the endocytic function of the coelomocytes was unimpaired (Figure 3; Supplementary Figure S1B).

The relative extent of the peptidergic release defects in *unc-10* and *cla-1L* mutants closely parallels their previously reported synaptic vesicle (SV) exocytic defects, which are more pronounced in *unc-10* mutants (Krout et al., 2023). This was partly attributed to a more severe reduction in the synaptic expression of UNC-2, voltage-gated calcium channels, in *unc-10* mutants (Krout et al., 2023; Oh et al., 2021). In support of this, a comparison of evoked SV release in 1 mM versus 5 mM extracellular Ca^2+^ showed that *unc-10* mutants exhibit a steeper drop-off in calcium-dependent release than *cla-1L* mutants (Figure 3; Supplementary Figure S1C, D) (Stigloher et al., 2011). Given that DCV release requires higher calcium concentrations than SV release, this likely contributes to the severe defects in peptide secretion in *unc-10* mutants (Voets et al., 2001).

In summary, both *cla-1L* and *unc-10* mutants exhibit multiple ultrastructural abnormalities. The relative abundance of irregular vesicles observed in *cla-1L* and *unc-10* mutants mirrors the extent of RAB-5 accumulation in each, suggesting that these vesicles may represent immature endosomes. These data support previous genetic evidence implicating *cla-1L* in endosomal formation and suggest *unc-10* may also be involved in the endosomal pathway (Xuan et al., 2023).

### Lysosomal organelles aggregate in the cell bodies of *cla-1L* and *unc-10* mutants

**FIGURE 4:**
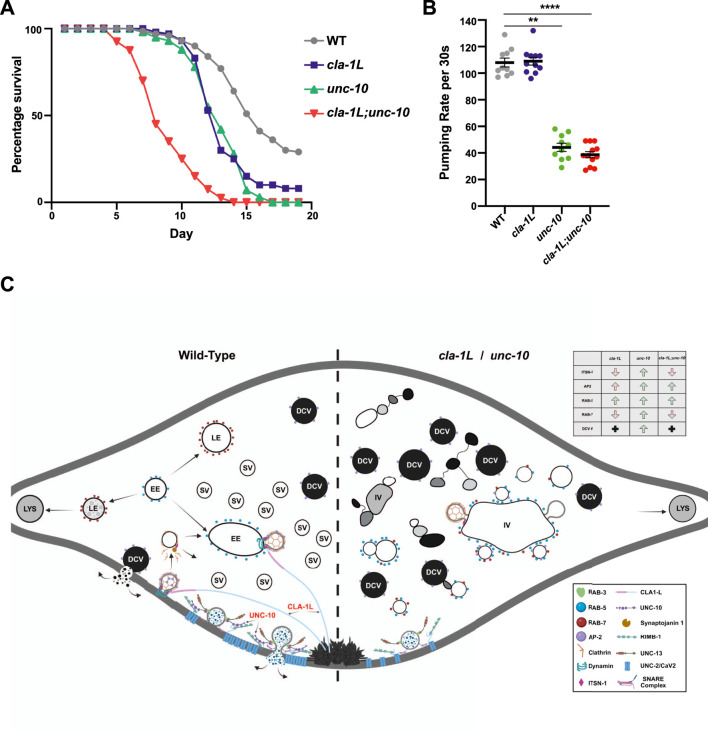
Loss of CLA-1L and UNC-10 lead to shortened lifespan with a summary of underlying synaptic defects. **(A)** Graph displaying the percentage survival per day of WT and cla-1L, unc-10 and cla-1L; unc-10 mutants. **(B)** Pharyngeal pumping rates measured per 30 s. **(C)** Schematic summary of the combined analyses presented in the current study. A single synapse shown with WT synaptic processes depicted on the left and features of both cla-1L and unc-10 mutants depicted on the right. Box inset provides a key for proteins illustrated. Abbreviations: Lys- Lysosome; LE- Late Endosome; EE- Early Endosome; SV- Synaptic Vesicles; DCV- Dense Core Vesicle; IV- Irregular vesicle. Created in BioRender. Lai, P. (2025) https://BioRender.com/b6xm32v. Statistical analysis: One-way ANOVA with Kruskal–Wallis with Dunn’s test for multiple comparisons; ***p < 0.001, p < 0.0001 results showing mean and S.E.M.

We asked whether the apparent disruption of EE to LE maturation in *cla-1L*, and to a lesser extent *unc-10* mutants, could impact later degradative stages of the endo-lysosomal pathway. To investigate this, we examined the synaptic distribution of fluorescent-tagged CTNS-1 (CTNS-1:RFP), an integral lysosomal membrane protein. There was no change in the number of synaptic CTNS-1 fluorescent peaks, and though we observed a trend toward an increase of fluorescent peak intensity of CTNS-1 in both *cla-1L* and *unc-10* single mutants, only the *cla-1L;unc-10* double mutants exhibited a significant increase (Figure 4; Supplementary Figure S1A–C).

The above results might be expected as, though some lysosomes may form at synapses, current data asserts that the primary site of endo-lysosomal fusion and cargo degradation occurs in the neuronal cell bodies following retrograde transport (Gowrishankar et al., 2015; Gowrishankar et al., 2017; Johnson et al., 2016; Kulkarni and Maday, 2018). Therefore, we examined CTNS-1 in the cell bodies of *cla-1L*, *unc-10* and *cla-1L;unc-10* mutants. While the number of CTNS-1-positive clusters per cell body was unchanged in any of the mutants, the size of the clusters was significantly increased in both *cla-1L* and *unc-10* single mutants and the *cla-1L;unc-10* double mutants (Figure 4; Supplementary Figure S1D–F).

### Changes in the endo-lysosomal system correlate with a shortened lifespan in *cla-1L* and *unc-10* mutants

Defects in the endo-lysosomal system have been associated with several neurodegenerative and age-related disorders. Moreover, the endo-lysosomal pathway has been linked to lifespan regulation in *C. elegans*, with activation of the system extending lifespan and disruptions leading to reduced longevity. To investigate whether *cla-1L* and *unc-10* mutants affect longevity, we examined the lifespans of single and double mutants. We found that both *cla-1L* and *unc-10* single mutants exhibited shorter lifespans, worms dying approximately 3 days earlier than WT controls (Figure 4A). The *cla-1L;unc-10* double mutants were even more severely affected, dying about 7 days earlier than WT. If the reduction in lifespan is due to disruptions in endosomal maturation and trafficking, we hypothesized that *itsn-1(tm725)* null mutants would display a similar phenotype to *cla-1L* mutants. Consistent with this prediction, the lifespan of *itsn-1(tm725)* mutants was also shortened by 3 days (Figure 4; Supplementary Figure S1G).

To determine whether the shortened lifespans of *cla-1L* and *unc-10* mutants might reflect feeding deficits, we measured pharyngeal pumping rates. This assay uncovered significantly reduced pumping rates in both *unc-10* and *cla-1L:unc-10* mutants, whereas *cla-1L* mutants were unaffected (Figure 4B). Since both *cla-1L* and *unc-10* mutants exhibited similarly shortened lifespans, we can conclude that reduced feeding rates alone cannot account for this result. Furthermore, as the pumping rates of *cla-1L;unc-10* double mutants were no worse than *unc-10* single mutants, the more severe lifespan reduction cannot be attributed solely to feeding defects. It is, however, interesting to observe that *unc-10* mutants impact feeding and raises questions about which aspects of the mutant phenotype might be responsible, possibly reflecting changes in both synaptic and/or peptidergic signaling.

## Discussion

In this study we reveal novel phenotypes for mutations in the RIM-family proteins CLA-1 and UNC-10/RIM at *C. elegans* synapses. Specifically, we show that *cla-1L* exhibits abnormalities in the endo-lysosomal pathway, whereas *unc-10* mutants regulate not only synaptic transmission, as previously determined, but also profoundly impact peptide secretion. These perturbations were accompanied by additive reductions in lifespan.

### Endosomal maturation pathway

Despite the fact that *cla-1L* mutants have only a minor impact on synaptic transmission, at the EM level we observe a dramatic accumulation of irregular endosomal vesicles at NMJs, accompanied by increased Rab-5 expression and decreased Rab-7. Similar changes have been observed in mammalian Piccolo mutants, a protein that shares homology to CLA-1, through conserved C2 and PDZ domains. Like *cla-1L* mutants, loss of Piccolo results in mild neurotransmitter release defects, increased axonal Rab5 and reduced Rab7 expression, associated with an accumulation of abnormal endosome-like structures observed by EM. One additional phenotype observed in cultured hippocampal synapses of Piccolo mutant rats, was a profound synaptic vesicle recycling defect. This inability to recycle vesicles was linked to a deficit in Rab5-dependent formation of early endosomes required to recycle SVs, a phenotype that could be rescued by expressing a mutant form of Rab5(Q79L) that remains in the activated GTP-bound state (Gillooly et al., 2003; Ackermann et al., 2019). The mechanism by which Piccolo regulates Rab5 has been extensively studied; this regulation occurs through a Zinc Finger–mediated interaction with Pra1 (Prenylated Rab Acceptor 1), which recruits Rab5 to early endosomes (EEs) (Ackermann et al., 2019; Fenster et al., 2000). Rab5 is then activated by GEFs, and Rab5–GTP recruits EEA1 and ITSN-1 to drive endosomal fusion and synaptic vesicle (SV) recycling. In Piccolo mutants, impaired Pra1 recruitment limits EE formation and SV recycling, defects that can be rescued by expression of the Piccolo Zinc finger or ITSN-1 overexpression (Ackermann et al., 2019).

To what extent might CLA-1L have conserved functional roles with Piccolo? While there is no evidence that *C. elegans* has a Pra1 homolog, and CLA-1L lacks a zinc finger domain, it could act in a similar manner to target RAB-5 to endosomes for subsequent activation through different protein interactions. The presence of multiple PxxP motifs in the CLA-1L N-terminal provides opportunities for interactions with SH3 domain containing proteins that could lead to deficits in Rab-5 recruitment or activation thereby indirectly hampering ITSN-1 targeting, and/or CLA-1L could directly recruit ITSN-1. This model is supported by previous evidence that the perisynaptic N-terminal of CLA-1L colocalizes with the ITSN-1 binding partner AP-2 in WT animals (Xuan et al., 2023). In contrast to CLA-1L, UNC-10 likely has only a minor role in the localization of ITSN-1, potentially as a secondary consequence of reduced neurotransmitter release which would presumably decrease SV recycling.

The increase in RAB-5 peak intensity and the associated reduction in RAB-7 levels indicates that CLA-1L is necessary for endosomal development and maturation. The additional increase in somatic CTNS-1 cluster size in the *cla-1L* and *unc-10* single and double mutants supports the idea that early disruptions in the endocytic pathway have consequences for the subsequent degradation of cargo required for normal cellular homeostasis. Mutants with autophagy deficits and endosomal axonal trafficking also accumulate lysosomes (de Araujo et al., 2020; Johnson et al., 2016). Therefore, the increase in somatic CTNS-1 clustering we observe in the *cla-1L* and *unc-10* mutant neurons could result from a decrease in lysosome degradation resulting from defects in endosomal maturation as well as trafficking of lysosomal hydrolases, and other lysosomal proteins, necessary for efficient degradation (de Araujo et al., 2020; Johnson et al., 2016; Majumder et al., 2022). Alternatively, when synaptic endosomes fail to mature into late endosomes, undegraded material accumulates at synapses, increasing the demand for compensatory lysosomal biogenesis in the soma, resulting in lysosome clustering.

### Pleiomorphic vesicle accumulation

Endosomal dysfunction is the earliest known pathobiology observed in Alzheimer’s Disease, wherein patient neurons accumulate endosomal vesicles (Cataldo et al., 2000; Nixon et al., 2005; Yu et al., 2004). Accumulation of endosomal vesicles at synapses can result from defective neurotransmitter release, dysfunctional endosomal and autophagic pathways as well as impaired lysosomal degradation (Cataldo et al., 2000; Nixon et al., 2005; Yu et al., 2004). Roles for CLA-1L have been previously established in synaptic function and localization of the key autophagy protein ATG-9. Here we place CLA-1L in the endosomal maturation pathway, a finding consistent with previous genetic evidence that ATG-9 missorting observed in *cla-1L* is exacerbated by *itsn-1* and *ehs-1* mutations (Xuan et al., 2023). Thus, loss of CLA-1L can result in the disruption of both the endo- and autophagic lysosomal pathways. At the NMJs, *cla-1L* mutants lead to an accumulation of pleomorphic vesicles, a phenotype shared with *itsn-1* mutants (Wang et al., 2008), along with changes in endosomal markers. This is in contrast to the ultrastructure of AIY neurons, which in the absence of CLA-1L, do not exhibit obvious morphological abnormalities at the EM level, yet accumulate ATG-9 in aberrant foci that colocalize with clathrin (Xuan et al., 2023). AIY is a highly specialized thermosensory neuron that has extraordinarily large synapses with thousands of SVs, which presumably are required for its function. This contrasts sharply with the small *en passant* NMJs studied here. The comparative differences in *cla-1L* mutant ultrastructural phenotypes, suggests that while CLA-1L can regulate both endo-lysosomal and autophagic pathways, these may be differentially utilized to meet the requirements of specific neurons.

### Dense core vesicles and neuropeptide release

In contrast to *cla-1L* mutants, we found that *unc-10* mutant NMJs accumulate DCVs, which correlates with decreased peptide secretion. These observations agree with recent literature demonstrating an essential role for mammalian UNC-10 homolog, Rim, in DCV release from cultured mouse hippocampal neurons (Persoon et al., 2019). In that study, synaptic DCV numbers trended higher, but more convincingly were found to be defective in stimulus-evoked fusion using optical reporters. Rim was also shown to co-transport with DCVs via Rab-3 interactions, providing a mechanism to target DCVs and recruit Munc-13 to release sites. More recent details have emerged that demonstrate (M)UNC-13 and Rim act synergistically in both DCV and SV fusion through different Rim and (M)unc-13 membrane-binding domains (Murphy et al., 2025). We recently reported that *unc-10* mutants have a significant decrease in presynaptic localization of UNC-13, possibly reflecting the disruption of Rim-dependent interactions found to protect Munc-13 from proteasomal degradation (Krout et al., 2023). These studies, and our previous finding that *unc-10* mutants have reduced UNC-2 calcium channel expression, provide an explanation for the DCV accumulation observed in *unc-10* mutants (Krout et al., 2023; Oh et al., 2021). Interestingly, a BioRxiv study, found the alpha subunit of AP-2 localizes to DCVs in human dorsal root ganglion neurons (Joshi et al., 2025). This finding provides a possible explanation for the increase in APT-4 (the AP-2 alpha subunit) and AP-2-associated Intersectin expression levels observed in the *unc-10* mutants–as a consequence of the dense core vesicle accumulation.

### Lifespan

Loss of either CLA-1L or UNC-10 alone result in premature death, and the combinatorial loss of the two proteins exacerbated this lifespan phenotype. Piccolo mutant mice also exhibit increased postnatal mortality compared to their WT and heterozygous littermates (Mukherjee et al., 2010). In a *C. elegans* study investigating longevity, lifespan was not well correlated with the severity of synaptic release defects, paralyzed *unc-13* and *unc-64*/syntaxin mutants actually living longer, whereas *unc-18* mutants with equally devastating release defects exhibited a normal lifespan, while other synaptic mutants resulted in shorter lifespans (Shen et al., 2007). These data suggest that the decreased lifespans we observed in the *cla-1L* and *unc-10* single and double mutants may result from roles beyond those in neurotransmitter release, possibly involving the endosomal and neuropeptide release defects detected here. Autophagy mutants, including Atg9, also have reduced lifespans across species (mammals, *Drosophila* and *C. elegans*) (Choi et al., 2024; Kojima et al., 2015; Wen et al., 2017). Given that CLA-1L has been implicated in ATG-9 sorting and autophagosome formation, it appears possible that both endosomal and auto-lysosomal pathways may contribute to longevity via their respective roles in neuronal health. Consistent with this proposition Piccolo has been shown to regulate neuronal integrity through both endo-lysosomal and autophagy pathways (Waites et al., 2013).

## Methods

### Confocal microscopy

Worms were picked as L4 stage larvae and imaged the following day as young adults. Approximately ten worms were placed in a drop of M9 on 2% agarose pads (in M9 buffer containing 10 mM sodium azide (NaN_3_)), on a glass slide, rotated with an eyelash hair to position the dorsal nerve cord closest to the objective, before covering with a glass coverslip. Nerve cord images were collected on an Olympus Fluoview FV10i inverted laser scanning confocal microscope with the 60X (NA 1.35) oil immersion lens and optical zooming to a total magnification of ×120. The same imaging parameters were used for each genetic background of the same fluorescent marker. All neuronal fluorescent markers were expressed in a subset of dorsal projecting cholinergic motor neurons using the *Punc-129* promoter. Wildtype strains were always imaged on the same day as mutant strains. Fluorescent analysis was conducted using NIH FIJI/ImageJ software in which max projections were created from obtained z-stacks, nerve cords were straightened, and all images were subjected to background subtraction with a rolling ball radius of 50. Fluorescent levels were extracted from a 40 µm line along the nerve cord and peaks were identified from the Plot Profile data using the Peak Finder function in Matlab. Statistical analysis was conducted in Prism (GraphPad) using one-way ANOVA with Dunn’s multiple comparison test.

### Peptide secretion assay

Dorsal nerve cords (DNCs) expressing NLP-21::GFP were imaged at ×120 magnification and individual coelomocytes in the tail region were imaged at a total magnification of ×480. Maximal projections of the DNC were generated in Fiji/ImageJ and peak fluorescence intensities were measured using peak finder in Matlab. For coelomocytes NLP-21::GFP uptake, summed projections were generated, and average fluorescence intensity of each circumscribed coelomocyte was measured, using Fiji/ImageJ.

### Coelomocyte endocytosis assay

Young adult worms were immobilized on agar pads using cyanoacrylic glue (Histoacryl Blue) before microinjecting Texas Red-BSA (1 mM) into the pharyngeal pseudocoelom, then kept in a drop of M9 for 20 min before exchanging with 20 mM NaN_3_ to arrest coelomocyte endocytosis. Coelomocytes were imaged at 480X and summed projections from z-stacks were generated and quantified in ImageJ.

### Electron microscopy

High pressure freeze fixation and freeze substitution (HPF/FS) was used to prepare strains as previously described (Liu et al., 2021; Weimer, 2006). Briefly, twenty to thirty young adult worms were placed in specimen chambers filled with *E. coli* and frozen at −180 °C, using liquid nitrogen under high pressure (Leica HPM 100, Leica, Oberkochen, Germany). Freeze substitution (Leica AFS2) was conducted using the following program: −90 °C for 107 h with 0.1% tannic acid followed by 2% OsO_4_ in anhydrous acetone, incrementally warmed at a rate of 5 °C/h to −20 °C, and kept at −20 °C for 14 h before increasing temperature by 10 °C/h to 20 °C; samples were then infiltrated with 50% Embed 812/acetone (EMS) for 4 h, 90% Embed 812/acetone for 18 h, and 100% Embed 812 for 5 h; samples were then embedded in Embed 812 and incubated for 48 h at 65 °C (Liu et al., 2021). Ultra-thin (40 nm) serial sections were acquired using an Ultracut 6 (Leica) and collected on formvar-covered, carbon-coated copper grids (EMS, FCF2010-Cu). Sections were post-stained with 2.5% aqueous uranyl acetate for 4 min, followed by Reynolds lead citrate for 2 min (Liu et al., 2021). Images were obtained using either a JEOL JEM1220 or JEM-1400F transmission electron microscope, operating at 80 kV. Micrographs were acquired using one of the following cameras: Gatan Es1000W 11 MP CCD, AMT NanoSprint1200-S CMOS or BioSprint 12M-B CCD Camera with AMT software (Version 7.01). Synapses at the NMJ of the ventral nerve cord were identified based on established synaptic morphology (White et al., 1986). Sections containing a dense projection (DP), as well as two flanking sections on either side of the DP, were analyzed blinded to genotype using NIH FIJI/ImageJ software. ROI data from FIJI were quantified using publicly available Matlab scripts written by the Jorgensen Lab (Watanabe et al., 2020). Total data analyzed: WT = 83 profiles from 12 synapses; *cla-1L* = 74 profiles from 10 synapses; *unc-10* = 64 profiles from 9 synapses; *cla-1L;unc-10* = 75 profiles from 11 synapses. Values were imported to Prism (GraphPad) for statistical analysis using One-way ANOVA with Tukey *post hoc* analysis, or Kruskal–Wallis with Dunn’s test, for multiple comparisons.

### Electrophysiology

Electrophysiological methods were as previously described (Richmond et al., 1999). Briefly animals were immobilized with a cyanoacrylic glue, and a lateral incision was made to expose the ventral neuromuscular synapses. Whole-cell voltage-clamp recordings from body wall muscles were made at −60 mV holding potential using a HEKA EPC-9 patch-clamp amplifier using Patchmaster software (HEKA). The extracellular solution contained: 150 mM NaCl, 5mMKCl, either 5 or 1 mM CaCl_2_,1mM MgCl2, 10 mM glucose and 15 mM HEPES, adjusted pH to 7.4 and 330 mOsm. The recording pipette solution contained: 120mM KCl, 20 mM KOH, 4mM MgCl_2_, 5 mM N-tris (hydroxymethyl) methyl-2- aminoethane-sulphonic acid), 0.25 mM CaCl_2_, 4 mM NaATP, 36 mM sucrose, 5 mM EGTA, pH 7.2 and 315 mOsm. Subsequent analysis and graphing were performed using Igor Pro (Wavemetrics). All statistically derived values are plotted as mean ± S.E.M.

### Lifespan assay

Ten adult worms from each strain were bleached, their eggs allowed to hatch and mature to L4 stage at which point worms were plated on each of six plates for a total of 60 worms assayed for each strain. Worms were screened daily to determine survival, and surviving worms were moved to fresh plates each day. Animals were considered dead after 3 gentle head taps with a worm pick resulted in no movement. Worms were assayed for a total of 21 days. Percentage of worms alive for each day of the assay was plotted using Prism (GraphPad).

### Pharyngeal pumping assay

All animals used for the pumping assay were day 1 adults (24 h post L4). Approximately 10 animals for each genotype were placed individually on bacteria seeded worm plates, and pharyngeal pumping rates were counted for 30 per worm.

### Strains

All worm strains were raised at 20 °C on NGM plates seeded with OP50 *E. coli* as a food source.

The following mutant strains were obtained from the CGC: N2 Bristol strain (wildtype reference), *cla-1(ok560)*IV, *unc-10(md1117)*X and *itsn-1(tm725)X*. The following fluorescently tagged protein strains are from previously published studies, are integrated and were verified by PCR (Ch’ng et al., 2008; Edwards et al., 2009; Edwards et al., 2013; Sieburth et al., 2005). *C. elegans* strain names are representative of fluorescent label position with N-terminal tags coming before the protein name and C-terminal tags following. Joshua Kaplan provided *nuIs214* [*unc-129p::itns-1*::GFP + *myo-2p*::GFP], which was crossed into *cla-1(ok560)*-SY1726, *unc-10(md1117)*-SY1812, and *cla-1(ok560);unc-10(md1117)-*(SY 1813). Joshua Kaplan also provided *nuIs184* [*myo-2p*::GFP; *unc-129p*::APT-4:GFP, which was crossed into *cla-1(ok560)-*SY1722, *unc-10(md1117)*-SY1814 and *cla-1(ok560);unc-10(md1117)-*(SY 1815). Kenneth Miller provided: *ceIs59* [*unc-129p*::YFP:*rab-5; ttx-3p*::RFP], which was crossed into *cla-1(ok560)-*SY1816, *unc-10(md1117)*-SY1817 and *cla-1(ok560);unc-10(md1117)-*(SY 1818), *ceIs54* [*unc-129p::CFP::rab-7, ttx-3p::RFP, unc-129p::nlp-21::Venus*], which was crossed into *cla-1(ok560)-*SY1819, *unc-10(md1117)*-SY1893 and *cla-1(ok560);unc-10(md1117)-*(SY 1894) and *ceIs56* [*unc-129p*::CTNS-1a:RFP; *unc-129p::nlp-21*::Venus; *ttx-3p*::RFP], which was crossed with *cla-1(ok560)-*SY1789, *unc-10(md1117)*-SY1830 and *cla-1(ok560);unc-10(md1117)-*(SY 1831).

## Data Availability

The original contributions presented in the study are included in the article/Supplementary Material, further inquiries can be directed to the corresponding author.
