# Supplementary material for: Differential roles for CLA-1L and UNC-10 in endosomal maturation and peptide release at C. elegans synapses impacting lifespan
Source: Front Mol Biosci. 2026 Jan 8;12:1675073. doi: 10.3389/fmolb.2025.1675073 (PMC12823496; doi:10.3389/fmolb.2025.1675073)
Supplement: Supplementary file 1 [file DataSheet1.pdf]

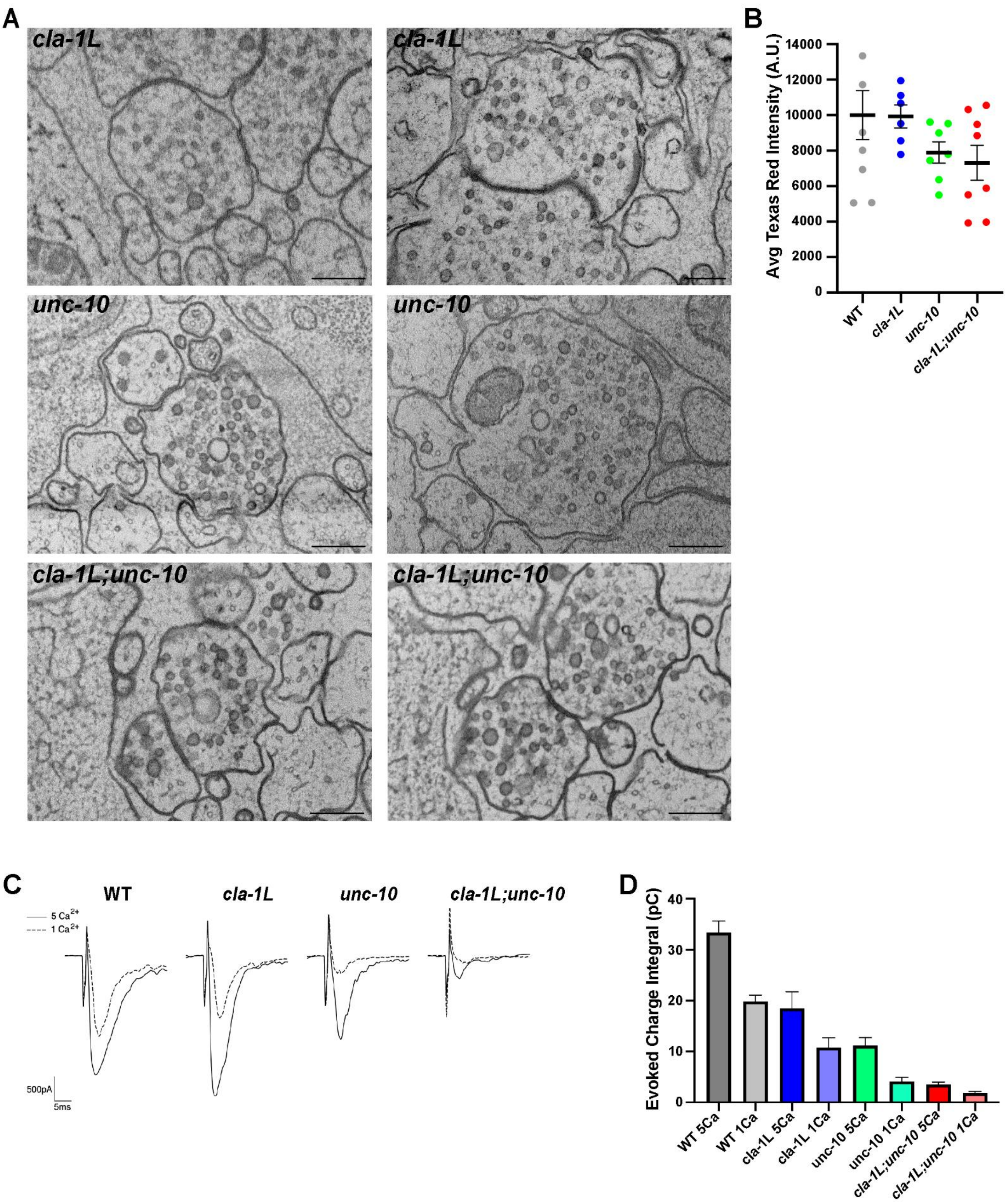

Figure 3 Supplement 1

Figure 3 Supplement 1.

A) Representative electron micrographs of NMJ synapses showing accumulation of irregular vesicles in *cla-1L* (top), *unc-10* (middle) and *cla-1L;unc-10* (bottom) mutant animals. Scale bar 200nm. **B)** Quantification of WT, *cla-1L*, *unc-10* and *cla-1L;unc-10* mutants summed projections from z-stacks of Texas Red BSA uptake into Coelomocytes 20mins post-injection **C)** Representative traces of electrophysiological recordings from *C. elegans* NMJs. **D)** Quantification of the charge integral for $5Ca^{2+}$ and $1Ca^{2+}$ in WT, *cla-1L* and *unc-10* and *cla-1L;unc-10* double mutants.

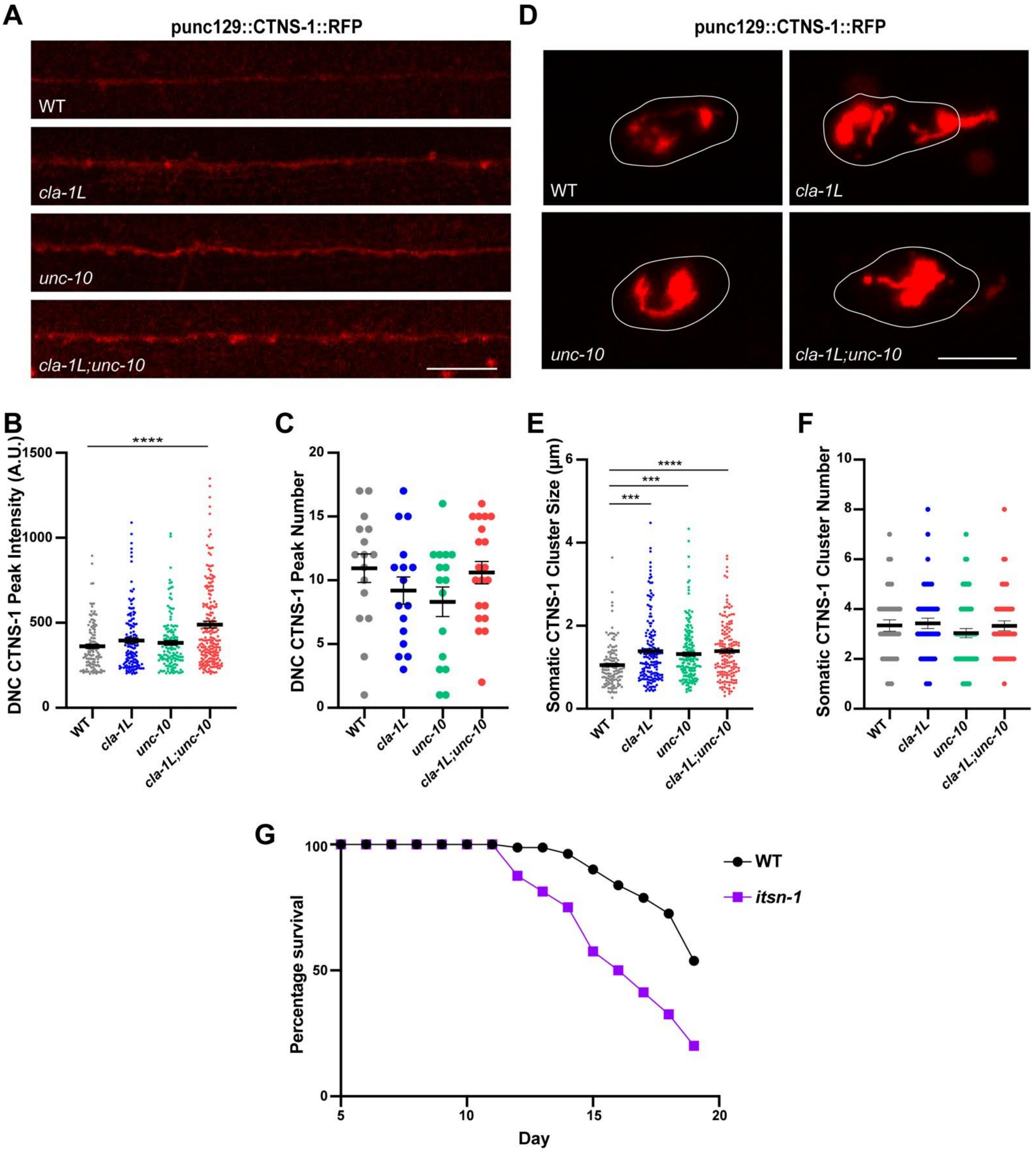

Figure 4 Supplement 1

Figure 4 Supplement 1.

A) Representative images of dorsal nerve cords of WT and *cla-1L*, *unc-10* and *cla-1L;unc-10* mutant animals expressing CTNS-1::RFP. Scale bar 10µm. **B-C)** Graphs displaying quantitative analysis of B) fluorescent peak intensity and C) peak number of CTNS-1::RFP in WT and mutant animals. **D)** Representative images of cholinergic neuronal cell bodies of WT and *cla-1L*, *unc-10* and *cla-1L;unc-10* mutant animals expressing CTNS-1::RFP, cell bodies are circled in white. Scale bar 10µm. **E-F)** Graphs displaying quantitative analysis of E) CTNS-1::RFP cluster size and F) CTNS-1::RFP cluster number in WT and mutant animals. **G)** Graph displaying the percentage survival per day of WT and *itsn-1* mutants. Statistical analysis: One-way ANOVA with Kruskal-Wallis with Dunn's test for multiple comparisons; *** $p < 0.001$, **** $p < 0.0001$ results showing mean and S.E.M.
